# Incidental finding of central pontine myelinolysis on [^18^F]FDG PET/CT

**DOI:** 10.1007/s00259-026-07923-1

**Published:** 2026-05-20

**Authors:** Teresa Antonia Kiener, Tobias Salzmann, Gundula Rendl, Mohsen Beheshti, Christian Pirich

**Affiliations:** Department of Nuclear Medicine and Endocrinology, University Hospital Salzburg, Paracelsus Medical University Salzburg, Müllner Hauptstraße 48, Salzburg, 5020 Austria

A 56-year-old man with a history of chronic alcohol abuse was admitted to the intensive care unit with hypotension and severe hyponatremia, with a sodium level of 114 mmol/L. After discontinuation of vasopressors and normalization of sodium levels, he was transferred to the general ward. Subsequently, Staphylococcus aureus bacteremia was diagnosed, and transesophageal echocardiography showed mild aortic valve thickening, suggestive of infective endocarditis. Intravenous cefazolin was started and the general condition of the patient slowly improved.

One week after admission, the patient developed mild ataxia und a multidirectional nystagmus. At the same time, [^18^F]FDG PET/CT was performed to exclude additional infectious foci. The study revealed no lesions indicative of infection. However, intense focal [^18^F]FDG uptake was seen in the pons with a SUVmax of 12.6 (Panel A - C, arrows), suggestive of central pontine myelinolysis [1, 2]. Retrospective review of electrolyte levels showed a rapid rise in sodium levels directly after admission, with an increase of 13 mmol/L within the first 24 h, supporting the suspected diagnosis. Magnetic resonance imaging of the brain was performed one day after [^18^F]FDG PET/CT and confirmed central pontine myelinolysis with a T2-hyperintense signal in the central pons, forming a characteristic “trident” sign on axial images (Panel D, arrowhead) with restricted diffusion (Panel E, arrowhead) [3]. Since the clinical presentation and imaging findings were consistent with central pontine myelinolysis, the consulting neurologists decided against further diagnostic workup and recommended supportive therapy along with physical rehabilitation. Over the subsequent weeks, the patient’s symptoms resolved completely.

Central pontine myelinolysis, or osmotic demyelination syndrome, may occur with a latency of up to one week after overly rapid correction of hyponatremia (typically following correction of more than 10 mmol per liter per 24 h) [4]. Clinical presentation might range from confusion and movement disorders to dysarthria, dysphagia and varying degrees of paresis to seizures, coma, or even locked-in syndrome [5]. 

This case underscores the potential diagnostic value of [^18^F]FDG PET/CT in the assessment of suspected central pontine myelinolysis following overcorrection of chronic hyponatremia, even in patients with only mild clinical symptoms.

**Figure:**
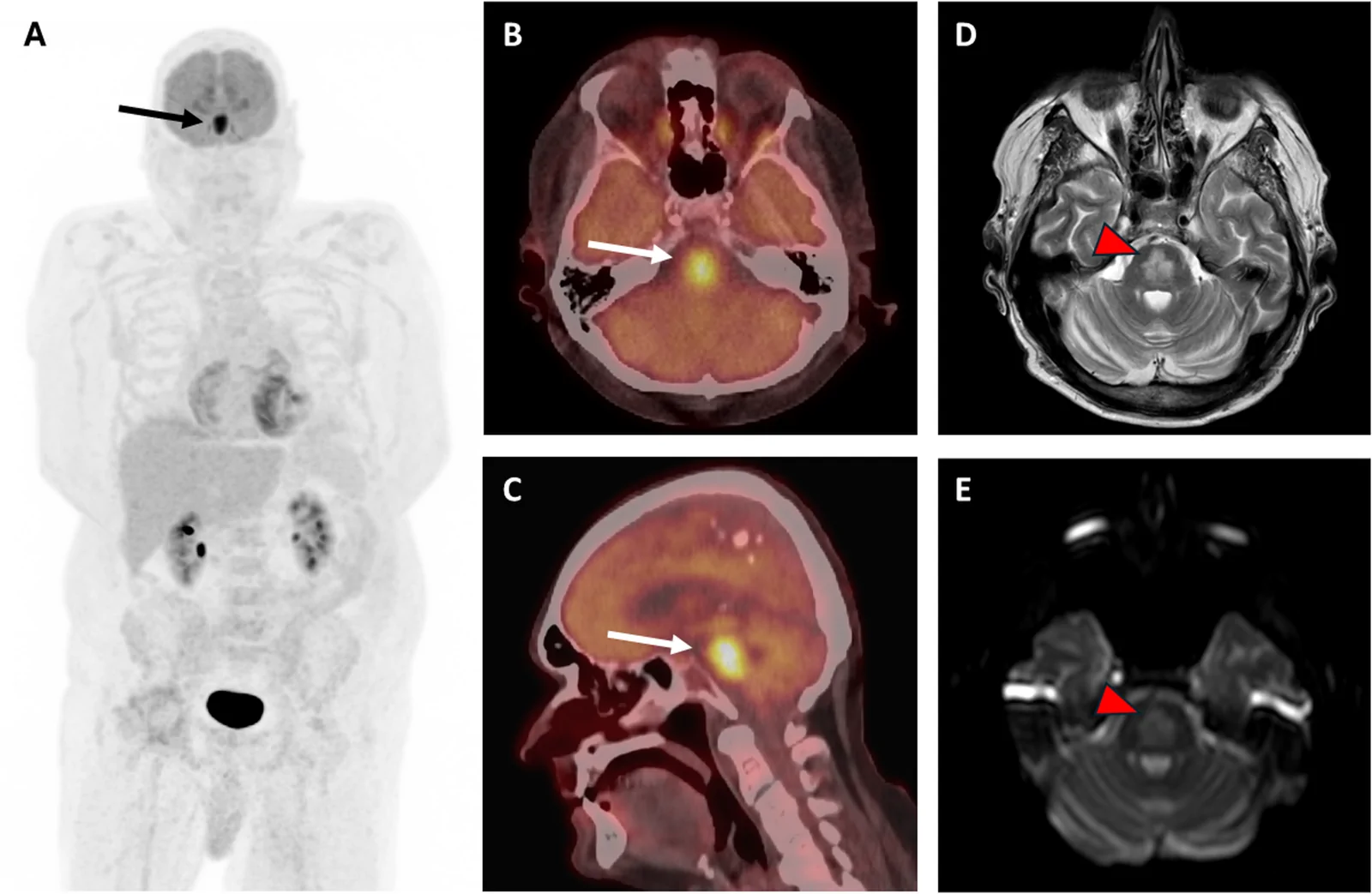

## Data Availability

The data analysed for this case study are available from the corresponding author on reasonable request.
